# Supplementary material for: Correcting the reproduction number for time-varying tests: A proposal and an application to COVID-19 in France
Source: PLoS One. 2023 Feb 27;18(2):e0281943. doi: 10.1371/journal.pone.0281943 (PMC9970098; doi:10.1371/journal.pone.0281943)
Supplement: S1 File — (PDF) [file pone.0281943.s001.pdf]

```
## R code to accompany the paper ``Correcting the Reproduction Number for Time-Varying Tests:
## a Proposal and an Application to COVID-19 in France''
## C. Baunez, M. Degoulet, S. Luchini, M. Pintus, P. Pintus, M. Teschl
## Revised version submitted to PLoS ONE on January 2, 2023

rm(list=ls())

tests.All # Cumulated cases
cases.All # Cumulated tests

##

nn <- length(cases.All) # Number of observations

eee     <- rep(0,nn) # Acceleration index
R.1     <- rep(0,nn) # Reproduction number
cases.T <- rep(0,nn) # Cumulated cases
tests.T <- rep(0,nn) # Cumulated tests

A.T.2 <- rep(0,nn) # Infectivity intensity
B.T.2 <- rep(0,nn) # Tests intensity

ff.r <- rep(0,nn) # Infectivity

for (i in 9:nn){
    # Normalized tests and cases
    tests   <- tests.All[1:i] / tests.All[i]
    cases   <- cases.All[1:i] / cases.All[i]
    # Acceleration index
    dcases  <- diff(cases)
    dtests  <- diff(tests)
    eee[i]  <- dcases[i-1] / dtests[i-1] # Acc. index
    # Infectivity
    dcases.A <- diff(cases.All) # daily cases
    ff.r[i] <- mean(dcases.A[(i-8):(i-1)]) # Infectivity
    # R reproduction number
    R.1[i]  <- dcases.A[i-1] / mean(dcases.A[(i-8):(i-1)])
    # Decomposition
    dtests.A <- diff(tests.All) # daily tests
    A.T.2[i]  <- mean(dcases.A[(i-8):(i-1)])/(cases.All[i]/i)
    B.T.2[i]  <- dtests.A[i-1]/tests.All[i]*i}


### Non parametric estimates

# Acc. Index

mod.ee <- loess(eee~seq(1,nn),span=0.1)
print(mod.ee)
predictionEE <- predict(mod.ee, newdata=seq(1,nn),se=TRUE)
ee.fit=predictionEE$fit

# Reproduction number

mod.rr <- loess(R.1~seq(1,nn),span=0.1)
print(mod.rr)
predictionRR <- predict(mod.rr, newdata=seq(1,nn),se=TRUE)
rr.fit=predictionRR$fit
```
