# Supplementary material for: Correcting the reproduction number for time-varying tests: A proposal and an application to COVID-19 in France
Source: PLoS One. 2023 Feb 27;18(2):e0281943. doi: 10.1371/journal.pone.0281943 (PMC9970098; doi:10.1371/journal.pone.0281943)
Supplement: S1 Appendix — (PDF) [file pone.0281943.s002.pdf]

Supporting information

Data Description and Computational Processing All the source data used in this study are available publicly from the web page “Données relatives aux résultats des tests virologiques COVID-19 SI-DEP” `https://www.data.gouv.fr/fr/datasets/donnees-relatives-aux-resultats-des-tests-virologiques-covid-19/` for France and from Our World in Data via `https://ourworldindata.org/coronavirus` for the five other countries, from which we extract the raw daily data for both the number of tests performed on a given day and the associated positive cases detected the same day from those tests, over the period from May 13, 2020, to October 26, 2022, for France, and over the period from May 13 to November 19, 2020, for the five other countries. In Table 1, we provide both the definition of the input data and the computational processing for the output variables that are used in the empirical analysis of Section .

For convenience, we also report in Table 2 the beginning and end dates for the main non-pharmaceutical interventions against COVID-19 that have been implemented in France.

Exponential Growth: an Illustrative Example We now illustrate the relationship between the acceleration index and the reproduction number when time is assumed, to ease derivation of results, to be continuous and when the number of cases grows exponentially over time, as usually assumed in epidemiological models, of SIR type and related for example. See for example equation (12) in [1] . Although typically ignored in the latter strand of literature, we introduce tests and we assume that they also grow exponentially. More formally, using the notation in the previous section,

Table 1. Summary of data description and computational processing

Name	Variable	Computation
Daily tests	d_t	raw data from SI-DEP and Our World in Data
Daily positive cases	p_t	raw data from SI-DEP and Our World in Data
Cumulated tests	D_t	$\sum_{\tau=1}^{t} d_\tau$
Cumulated cases	P_t	$\sum_{\tau=1}^{t} p_\tau$
Acceleration index	ε_t	$\frac{p_t/d_t}{P_t/D_t}$
Infectivity	$\hat{f}_t$	$\frac{1}{n+1}\sum_{s=0}^{n} p_{t-s}$
Reproduction number	$\hat{R}_t$	$p_t/\hat{f}_t$
Infectivity intensity	A_t	$\hat{f}_t/(\frac{1}{t}P_t)$
Test intensity	B_t	$d_t/(\frac{1}{t}D_t)$

Table 2. Dates for main non-pharmaceutical interventions in France

Name	Date begins	Date ends
First Lock-down	Mar 17, 2020	May 11, 2020
Curfews	Dec 15, 2020	Jun 20, 2021
Second Lock-down	Oct 30, 2020	Dec 15, 2020
Third Lock-down	Apr 3, 2021	May 2, 2021
Vaccination	Dec 27, 2020	still ongoing

suppose that the number of cases per unit of time is denoted by $p(t) = \alpha e^{\beta t}$ while the number of tests per unit of time is $d(t) = \gamma e^{\nu t}$, where the growth rates β and ν are assumed to be positive for the sake of illustration. The reproduction number is then constant over time, as we now show when the infection kernel is uniform, that is, if the weights w are constant and equal to $1/\delta$ to ensure that $w\int_0^\delta ds = 1$. Note that it is not difficult to show that the constancy of $\hat{R}$ holds for other infection distributions as well. The analog of equation (4) is as follows:

$$\hat{R}(t) = \frac{p(t)}{w\int_0^\delta p(t-s)ds} \tag{8}$$

which implies that, given $p(t) = \alpha e^{\beta t}$, one has:

$$\hat{R}(t) = \hat{R} = \frac{\beta\delta}{1-e^{-\beta\delta}} \tag{9}$$

It is easily seen that the reproduction number is the growth rate of daily cases (adjusted for the delay δ) only, since it obviously does not take into account tests. In fact, $\hat{R}$ is the growth rate of the denominator in equation (8), that is, what we note $\hat{f}$ in the main text.

Cumulated cases and tests are then noted $P(t) = \int_0^t p(\tau)\,d\tau$ and $D(t) = \int_0^t d(\tau)\,d\tau$, respectively. It is easy to derive, by straight integration, the expressions:

$$P(t) = \frac{\alpha}{\beta}\left(e^{\beta t}-1\right),\ D(t) = \frac{\gamma}{\nu}\left(e^{\nu t}-1\right) \tag{10}$$

It follows that our acceleration index is given, as function of time, by:

$$\varepsilon(t) = \frac{p(t)/P(t)}{d(t)/D(t)} = \frac{\beta}{\nu}\left(\frac{1-e^{-\nu t}}{1-e^{-\beta t}}\right) \tag{11}$$

From equation (11), one concludes that the acceleration index, which is an elasticity that measures the responsiveness of cases to tests, is essentially the *ratio of the growth*

rate of cumulated cases divided by that of cumulated tests, since $p(t) = dP(t)/dt$ and $d(t) = dD(t)/dt$. In addition, ε tends to the ratio of the growth rate of daily cases to that of daily cases β/ν. This means that the acceleration index tracks that ratio over time and converges to it eventually.

It follows that three cases occur. When $\beta = \nu$, that is, when both daily cases and daily tests grow at the exact same rate, then our acceleration index equals 1 at all dates. When the two growth rates differ, however, $\varepsilon(t)$ converges, when t goes to infinity, to the ratio of growth rates β/ν, independently of the scale parameters α and γ. As an illustrative example, suppose that $\beta > \nu$, so that positives grow faster than tests. Then the pattern of our acceleration index $\varepsilon(t)$ over time will have two regimes: it first grows almost linearly and eventually reaches the upper bound $\beta/\nu > 1$. Obviously, in that case both the daily positivity rate $p(t)/d(t)$ and the average positivity $P(t)/D(t)$ grow over time, and the latter quantity exceeds the former all the time so that acceleration prevails. The symmetric case when $\beta < \nu$ is easily adapted.

The property that the reproduction number is constant under our assumptions essentially means that the dynamics of the acceleration index is driven by that of $A(t)/B(t)$, which we now decompose to help understand how $\hat{R}$ and ε compare over time. It follows from the definition of A and B in equation (5) that:

$$A(t) = t\left(\frac{e^{\beta t} - e^{\beta(t-\delta)}}{\delta(e^{\beta t} - 1)}\right) \quad \text{and} \quad B(t) = \nu t\left(\frac{e^{\nu t}}{e^{\nu t} - 1}\right) \tag{12}$$

To further illustrate what happens in the theoretical case outlined above with $\beta > \nu$, let us take a numerical example. Suppose that the growth rate of cases β equals 20% while the growth rate of tests $\nu = 10\%$. In addition suppose that the delay parameter $\delta = 4$ so that the weight $w = 1/4$. Figure 2 illustrates how the acceleration index and the reproduction number, as well as the infectivity and test intensities, evolve over time in this particular example, with both functions $A(t)$ and $B(t)$ increasing with time t.

The exponential example is also useful to illustrate the formal relationship between viral speed and the acceleration index. To do that, let us now make an analogy with linear body motions to relate our indicators to speed and acceleration. If one think of the positivity rate as viral speed and define it as $s(t) = \frac{p(t)}{d(t)}$ then straightforward computations give $s(t) = \frac{\alpha}{\gamma}e^{(\beta-\nu)t}$. By analogy, viral acceleration could be defined as the derivative of viral speed, that is, $a(t) = s'(t) = \frac{\alpha}{\gamma}(\beta - \nu)e^{(\beta-\nu)t}$. Not surprisingly, then, it turns out that the *sign* of $a(t)$ indicates acceleration in the sense that a positive sign reveals acceleration (this happens when $\beta > \nu$ since speed goes up over time in that case) while a negative sign reveals deceleration (when $\beta < \nu$).

However, a drawback of $a(t)$ as a *measure* of acceleration is that it is scale-dependent since it depends on scale parameters α and ν, though their ratio. In other words, $a(t)$ depends on the absolute level of speed, that is, of the positivity rate. On the contrary, the acceleration index given by Equation (11) does not and is unit-free, since it is an elasticity. Note that a possible, also unit-free, alternative would be to define acceleration as the semi-elasticity $a(t)/s(t) = \beta - \nu$. However, since it relates the percentage change of positives case to the *absolute* change of tests, it is arguably less amenable to interpretation than the elasticity. It is easily shown that the semi-elasticity would be obtained if the average positivity rate would alternatively be defined as the average of the daily positivity rates up to date T, as opposed to the ratio of number of cumulated cases to the number of cumulated tests as in Equation (2) which leads to the acceleration index as an elasticity. Finally, note that the acceleration index equals one when viral speed stays constant over time.

Test-Controlling the Reproduction Number in Practice: Five Additional Countries In this appendix, we provide estimates for both the acceleration index and

the reproduction number, for Argentina, Austria, South Africa, South Korea and the United Kingdom, using the same method that is applied in Section for France and over a pre-vaccination period (May 13 to November 19, 2020). The public data source for the five countries comes from Our World in Data (see [2]). The resulting estimates, depicted in Figures 5 to 9 (that are hence directly comparable to Figure 3 for France), unambiguously reveal that the substantial bias due to time-varying tests, which is corrected by the acceleration index but not by the reproduction number, is not specific to France or to a particular period. In fact, the corresponding bias forces the reproduction number to either underestimate (Argentina over the whole period is a case in point) or overestimate viral acceleration, depending on the country and the period considered. This observation suggests that a similar bias should come as no surprise for other countries as well.

Fig 5. Argentina - Panel (a) Acceleration index (blue curve) vs reproduction number (green curve). Panel (b) Infectivity intensity (orange curve) vs test intensity (red curve). Panel (c) Kernel estimates with confidence bands (dashed lines). Panel (d) Daily tests (purple line) and infectivity function (black curve). Source: Our World in Data and authors' computations.

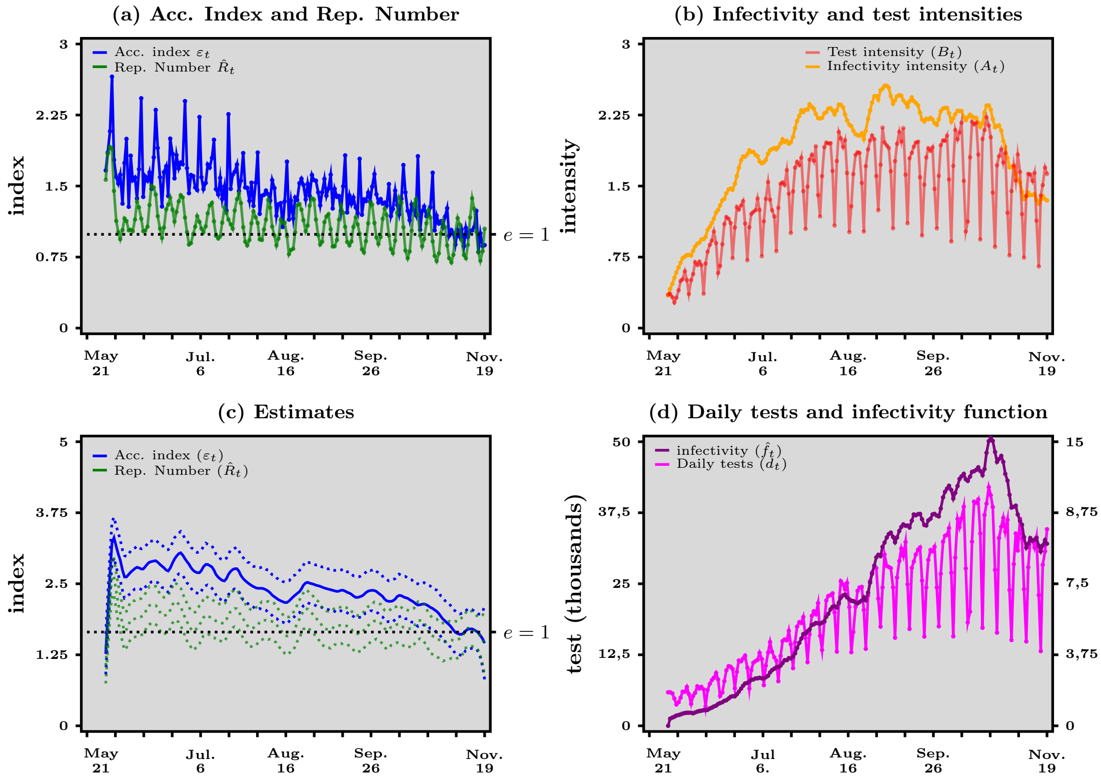

Fig 6. Austria - Panel (a) Acceleration index (blue curve) vs reproduction number (green curve). Panel (b) Infectivity intensity (orange curve) vs test intensity (red curve). Panel (c) Kernel estimates with confidence bands (dashed lines). Panel (d) Daily tests (purple line) and infectivity function (black curve). Source: Our World in Data and authors' computations.

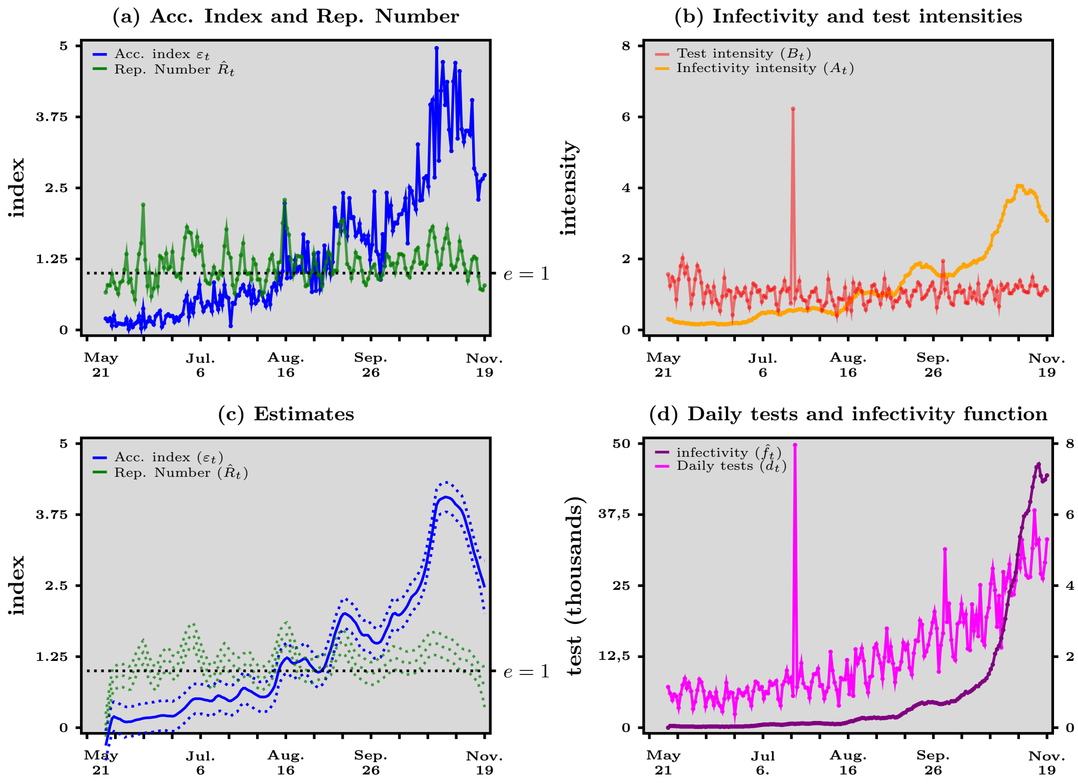

Economics, Toulouse School of Economics, for useful comments, as well as the Editor and both referees for suggestions that prompted us to improve the draft. This work was supported by the French National Research Agency Grant ANR-17-EURE-0020, and by the Excellence Initiative of Aix-Marseille University - A*MIDEX.

Fig 7. South Africa - Panel (a) Acceleration index (blue curve) vs reproduction number (green curve). Panel (b) Infectivity intensity (orange curve) vs test intensity (red curve). Panel (c) Kernel estimates with confidence bands (dashed lines). Panel (d) Daily tests (purple line) and infectivity function (black curve). Source: Our World in Data and authors' computations.

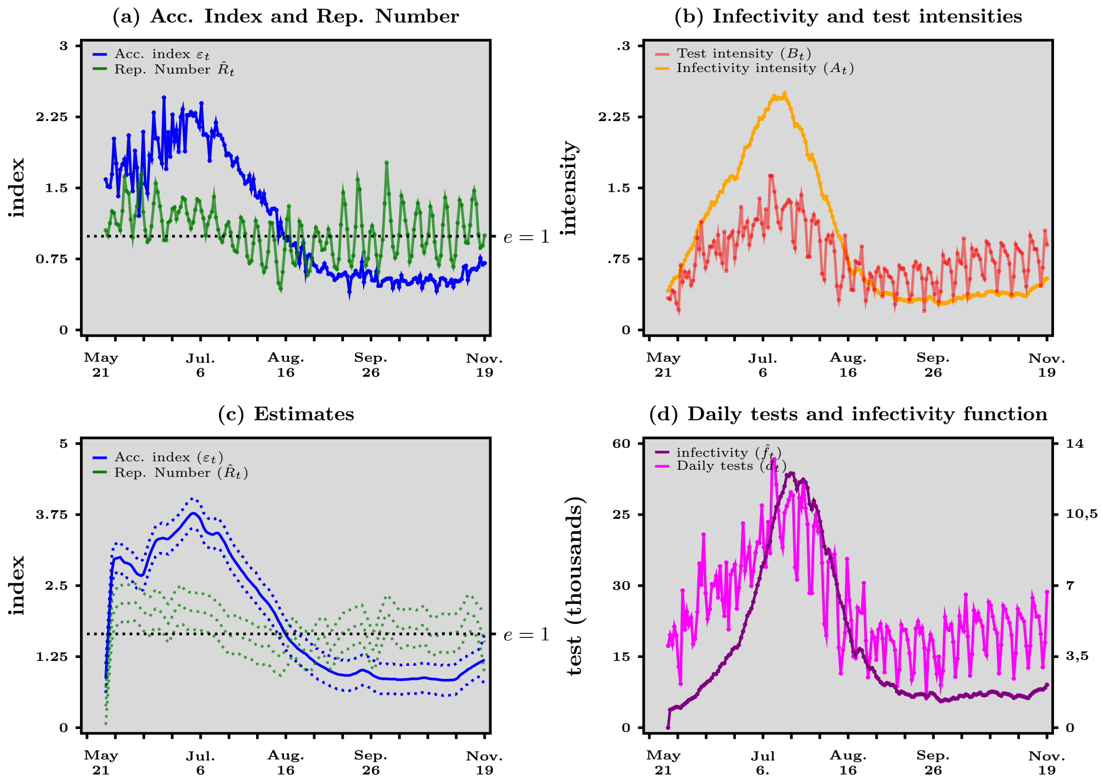

Fig 8. South Korea - Panel (a) Acceleration index (blue curve) vs reproduction number (green curve). Panel (b) Infectivity intensity (orange curve) vs test intensity (red curve). Panel (c) Kernel estimates with confidence bands (dashed lines). Panel (d) Daily tests (purple line) and infectivity function (black curve). Source: Our World in Data and authors' computations. Testing data for South Korea were incomplete: five dates were missing. Missing values were completed by a linear interpolation.

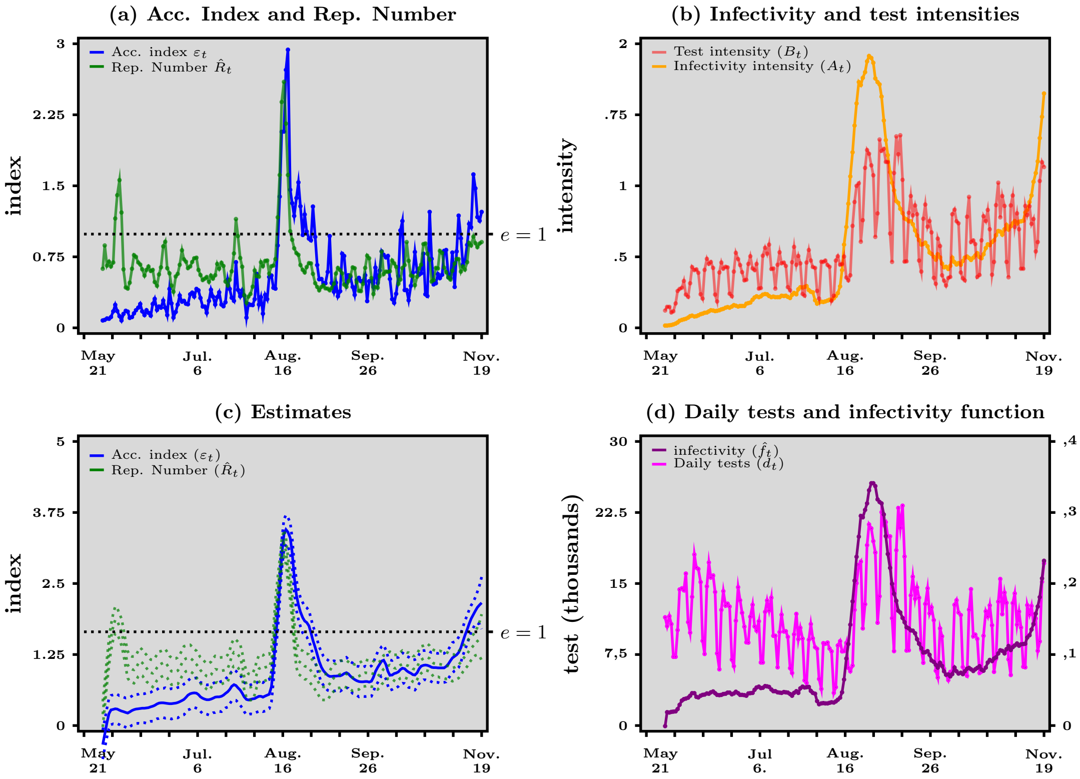

Fig 9. United Kingdom - Panel (a) Acceleration index (blue curve) vs reproduction number (green curve). Panel (b) Infectivity intensity (orange curve) vs test intensity (red curve). Panel (c) Kernel estimates with confidence bands (dashed lines). Panel (d) Daily tests (purple line) and infectivity function (black curve). Source: Our World in Data and authors' computations.

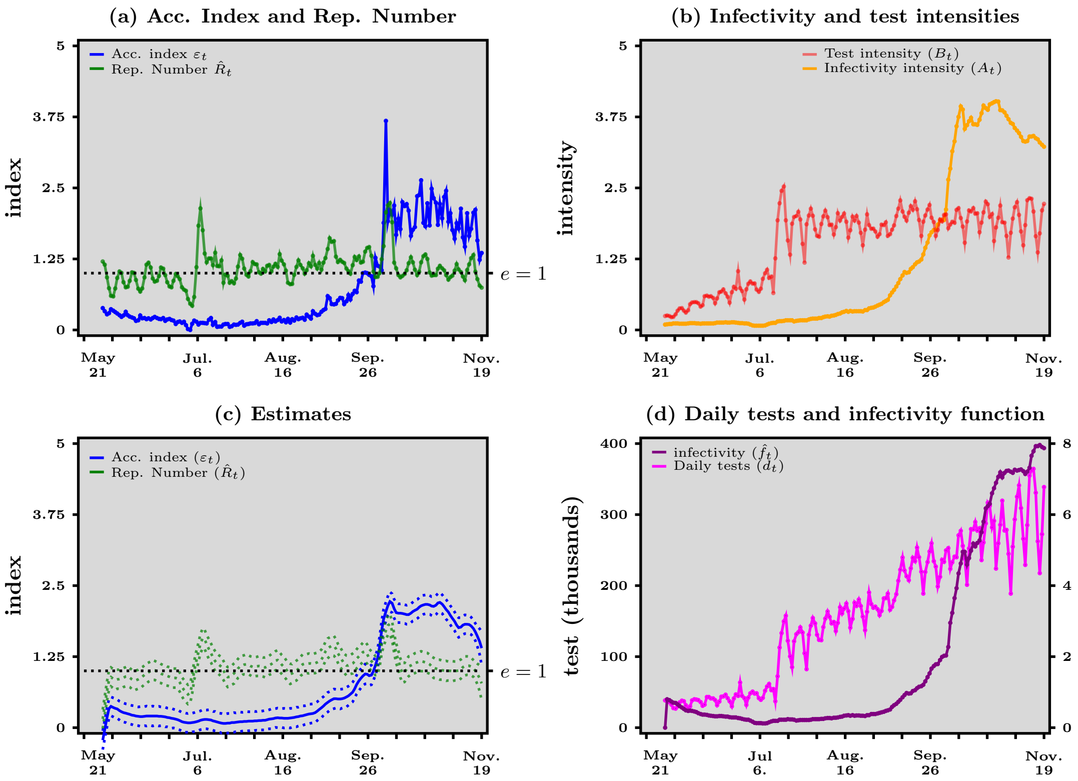

References

1. Fraser C. (2007). Estimating Individual and Household Reproduction Numbers in an Emerging Epidemic. *PLoS One*, 2:e758.

2. Ritchie, H., Mathieu, E. Rodés-Guirao, L., Appel, C., Giattino, C., Ortiz-Ospina, E., Hasell, J., Macdonald, B., Beltekian, D., Roser, M. (2020): Coronavirus Pandemic (COVID-19). Published online at OurWorldInData.org. Retrieved from: `https://ourworldindata.org/coronavirus` [Online Resource]
